# Longitudinal plasma proteomic analysis identifies biomarkers and combinational targets for anti-PD1-resistant cancer patients

**DOI:** 10.1007/s00262-024-03631-7

**Published:** 2024-02-13

**Authors:** Qiaoyun Tan, Ruyun Gao, Xiaomei Zhang, Jianliang Yang, Puyuan Xing, Sheng Yang, Dan Wang, Guibing Wang, Shasha Wang, Jiarui Yao, Zhishang Zhang, Le Tang, Xiaobo Yu, Xiaohong Han, Yuankai Shi

**Affiliations:** 1 Department of Medical Oncology, National Cancer Center/National Clinical Research Center for Cancer/Cancer Hospital, Chinese Academy of Medical Sciences & Peking Union Medical College, Beijing Key Laboratory of Clinical Study On Anticancer Molecular Targeted Drugs, Beijing, 100021 China; 2 State Key Laboratory of Proteomics, Beijing Proteome Research Center, National Center for Protein Sciences-Beijing (PHOENIX Center), Beijing Institute of Lifeomics, Beijing, 102206 China; 3 Clinical Pharmacology Research Center, Peking Union Medical College Hospital, State Key Laboratory of Complex Severe and Rare Diseases, NMPA Key Laboratory for Clinical Research and Evaluation of Drug, Beijing Key Laboratory of Clinical PK & PD Investigation for Innovative Drugs, Chinese Academy of Medical Sciences & Peking Union Medical College, Beijing, 100730 China

**Keywords:** Anti-PD1 therapy, IL-17A, Biomarker, Drug target, Proteomics

## Abstract

The response rate of anti-PD1 therapy is limited, and the influence of anti-PD1 therapy on cancer patients is unclear. To address these challenges, we conducted a longitudinal analysis of plasma proteomic changes with anti-PD1 therapy in non-small cell lung cancer (NSCLC), alveolar soft part sarcoma (ASPS), and lymphoma patients. We included 339 plasma samples before and after anti-PD1 therapy from 193 patients with NSCLC, ASPS, or lymphoma. The plasma proteins were detected using data-independent acquisition-mass spectrometry and customable antibody microarrays. Differential proteomic characteristics in responders (R) and non-responders (NR) before and after anti-PD1 therapy were elucidated. A total of 1019 proteins were detected using our in-depth proteomics platform and distributed across 10–12 orders of abundance. By comparing the differential plasma proteome expression between R and NR groups, 50, 206, and 268 proteins were identified in NSCLC, ASPS, and lymphoma patients, respectively. Th17, IL-17, and JAK-STAT signal pathways were identified upregulated in NR group, while cellular senescence and transcriptional misregulation pathways were activated in R group. Longitudinal proteomics analysis revealed the IL-17 signaling pathway was downregulated after treatment. Consistently, many proteins were identified as potential combinatorial therapeutic targets (e.g., IL-17A and CD22). Five noninvasive biomarkers (FLT4, SFTPB, GNPTG, F5, and IL-17A) were further validated in an independent lymphoma cohort (*n* = 39), and another three noninvasive biomarkers (KIT, CCL3, and TNFSF1) were validated in NSCLC cohort (*n* = 76). Our results provide molecular insights into the anti-PD1 therapy in cancer patients and identify new therapeutic strategies for anti-PD1-resistant patients.

**Supplementary Information:**

The online version contains supplementary material available at 10.1007/s00262-024-03631-7.

## Introduction

Immune checkpoint inhibitors (ICIs) targeting programmed death 1 (PD1) or programmed death-ligand 1 (PD-L1) have revolutionized the pattern of anti-tumor treatment with improved survival in multiple cancer types [1, 2]. However, a large portion of cancer patients cannot benefit from anti-PD1 therapy [3]. Previous genomic and transcriptomic studies have identified biomarkers for the efficacy prediction of anti-PD1 therapy. To date, only three markers were approved by the United States Food and Drug Administration (FDA) [4], including microsatellite instability (MSI)/mismatch-repair deficiency (dMMR) [5], tumor mutation burden (TMB) [6] in pancancer, and PD-L1 expression in non-small cell lung cancer (NSCLC) [7]. Moreover, the clinical predictive value of these biomarkers remains within the range of 20%–80% and is largely influenced by the assay used and cancer type [8–12]. Therefore, it is imperative to identify new biomarkers for anti-PD1 immunotherapy [13].

Proteomics technologies that can measure all proteins in clinical samples offer an alternative approach in elucidating the heterogeneous responses of cancer patients and discovering biomarkers for anti-PD1 therapy [14]. Compared to tissues, the detection of protein biomarkers is simple, noninvasive, and can be easily adapted in the clinic [15, 16]. In our previous study, we identified five autoantibody biomarkers for anti-PD1 therapy using high-throughput protein arrays [17]. In addition to autoantibodies, there are numerous plasma proteins that can be used as biomarkers in clinical diagnostics and prognosis prediction [18]. However, the detection of low-abundance proteins is particularly challenging for mass spectrometry (MS). To address this need, we developed an in-depth serum proteomic platform using DIA-MS and customizable antibody microarrays that can detect serum proteins covering 10–12 orders of abundance magnetite [19]. Using a similar strategy, Babačić et al. investigated serial plasma samples from 24 patients with metastatic melanoma and observed an increase in circulating PD1 during anti-PD1 therapy, as well as diverse immune plasma proteomic signatures in anti-PD1 responders [20]. However, the number of patients analyzed in that study is limited and thus requires validation using a larger cohort. Moreover, only one cancer type was investigated in the study, and the influence of PD1 therapy on different cancers is unclear.

In this study, we systematically analyzed the expression of the plasma proteome (*n* = 339) in a cohort of 193 NSCLC, alveolar soft part sarcoma (ASPS), and lymphoma patients longitudinally using our in-depth proteomic platform by integrating DIA-MS and antibody microarrays. The differentially expressed proteins between responders (R) and non-responders (NR) were identified by statistical analysis. Bioinformatics analysis further illustrated common and specific biological processes and signaling pathways that were altered in NSCLC, ASPS, and lymphoma cancer patients. In addition, biomarkers and combinational protein targets that could be potentially used to maximize therapeutic benefits for cancer patients were identified and validated in independent clinical cohorts.

## Materials and methods

### Plasma sample collection

We longitudinally collected serial plasma samples before and after treatment, including the 1st, 2nd, 3rd, or more than 3rd assessment time points. A total of 339 plasma samples were collected from August 2016 to March 2022 from 93 NSCLC patients (92 patients have pre-treatment samples), 12 ASPS patients, and 88 lymphoma patients (Table S1). Among NSCLC patients, six patients were EGFR mutation positive, none had ALK rearrangements, in 29 NSCLC patients with PD-L1 expression level available, and PD-L1 scores of TPS ≥ 50%, TPS 1 to 49%, and TPS < 1% were observed in 10, 11, and 8 patients, respectively. All blood samples were collected in EDTA tubes. After centrifugation at 16,000*g* at 4 °C for 10 min, the plasma was collected in a new tube and stored at − 80 °C until use.

Patient baseline characteristics including age at treatment start, gender, ECOG performance, and tumor stage are shown in Table S2. Three approved anti-PD1 antibodies including Sintilimab, Toripalimab, and Nivolumab were used. Treatment efficacy was evaluated by oncologists and radiologists according to clinical and radiological examination results. The clinical response was defined as complete response (CR), partial response (PR), stable disease (SD), or progressive disease (PD) based on Response Evaluation Criteria in Solid Tumours (RECIST) version 1.1 for ASPS, NSCLC, and International Working Group 2007 Criteria for lymphoma [21, 22]. Responder (R) patients were defined as patients with CR/PR or SD lasting ≥ 6 months. The non-responder (NR) group included patients who had PD on/before 6 months [23–25].

All experiments were conducted with the approval of the Research Ethics Committee and according to the Declaration of Helsinki.

### Screening of serum proteome using antibody microarrays

The preparation of antibody microarray and serum screening were performed as previously described [19]. Briefly, 10 μL of serum samples were diluted to 100 μL with phosphate-buffered saline (PBS pH 7.4) and then labeled by NHS-PEG4-Biotin (Thermo Fisher Scientific, MA, USA) at room temperature for 1 h. Excess biotin molecules were removed, and the biotinylated serum samples were diluted with 400 μL of PBS containing 5% milk (w/v). In parallel to biotin labeling, antibody microarrays were blocked with 500 μL of PBS with 5% milk (w/v) for 1 h at room temperature. After removing the milk, the microarrays were incubated with biotinylated serum for 2 h at room temperature, followed by washing with PBS containing 0.05% (w/v) Tween-20 (PBST). To detect bound proteins, the arrays were incubated with 2 µg/mL streptavidin–phycoerythrin (PE) (Jackson Immunoresearch, USA) for 1 h at room temperature in the dark and then washed with PBST. After centrifuging for 2 min at 1000*g*, the slides were scanned using a GenePix 4300A microarray scanner at a wavelength of 532 nm. BSA was used as negative control for protein detection by microarray.

### Measurement of serum proteome using DIA-MS

Peptide sample preparation and data-independent acquisition analysis were performed as previously described [26]. Briefly, 2 µL serum sample was diluted with lysis buffer containing 6 M urea (Sigma, USA). Next, the serum was reduced with 10 mM dithiothreitol (DTT) at 37 °C for 60 min and then alkylated with 500 mM iodoacetamide (IAA) at room temperature for 45 min in the dark. Proteins were sequentially digested with trypsin for 16 h at 37 °C. The tryptic peptides were acidified with 1% trifluoroacetic acid and desalted with a C18 desalination column according to the manufacturer’s protocol. The desalted peptide was dried under vacuum and dissolved in 20 μL of buffer containing 0.1% formic acid and 2% acetonitrile. Peptide concentrations were measured by Nanoscan (Analytik Jena AG, Jena, Germany). Approximately 1.5 µg of peptides were separated on a 30-min LC gradient using an analytical column (150 µm × 250 mm, 2 µm 200 Å C18 particles) and injected into a QE-HF mass spectrometer (Q Exactive HF Hybrid Quadrupole Orbitrap™, Thermo Fisher). The DIA acquisition scheme consisted of 45 fixed windows ranging from 350 to 1500 m/z. The windows were set for DIA acquisition as follows: 348–400, 400–424, 424–449, 449–465, 465–478, 478–489, 489–500, 500–514, 514–527, 527–540, 540–550, 550–559, 559–568, 568–579, 579–588, 588–599, 599–609, 609–621, 621–631, 631–641, 641–651, 651–663, 663–674, 674–686, 686–696, 696–708, 708–720, 720–733, 733–746, 746–760, 760–774, 774–788, 788–804, 804–820, 820–837, 837–856, 856–876, 876–898, 898–922, 922–949, 949–979, 979–1017, 1017–1064, 1064–1138, and 1138–1400 m/z. The resolution distribution of MS1 and MS2 was 60,000 and 30,000, respectively. A Spectronaut Pulsar X 12.0 (Biognosys, Schlieren, Switzerland) was used for identification and quantification. Finally, peptides FDR and proteins FDR were all set at 1% (FDR).

### Validation of biomarkers via enzyme-linked immunosorbent assay (ELISA)

To verify the prognostic value of biomarkers on the efficacy of NSCLC patients using immunotherapy, we collected plasma samples of an external cohort of 76 NSCLC patients. The ELISA kit we used was assessed from Novus, Proteintech, and Sino Biological. The detailed steps were strictly followed by the manufacturer’s instruction.

### Data analysis

We included 615 positive array-detected proteins and 509 MS proteins in the analyses, and all proteins were named by gene names. The testing data were normalized using the quantile method and log2 transformed. To investigate differentially expressed plasma proteins (DEPs) in pre-treatment samples between the R and NR groups and during treatment among the ASPS, NSCLC, and lymphoma patients, the Mann–Whitney *U* test was used, and differences with *p* < 0.05 were defined as statistically significant. The DEPs were clustered by the Hiplot platform (https://hiplot.com.cn).

Pathway and Gene Ontology (GO) enrichment analyses were performed by ClueGo in Cytoscape (version3.8.0) or omicsbean (http://www.omicsbean.cn/login/?next=/dashboard/). Protein drug targets were obtained from the Human Protein Atlas (HPA), comprehensive information was collected from the HPA and the Therapeutic Target Database (TTD) [27], and information on clinical trials was obtained from the clinical trial database (https:// clinicaltrials.gov/). Hierarchical clustering and principle component analysis (PCA) were implemented and plotted using the statistical language R (version 4.2.3). A Venn diagram was constructed using an online platform (http://bioinformatics.psb.ugent.be/webtools/Venn/). We used TIMER2.0 for analysis of immune infiltrates [28]. Survival curves were constructed using Kaplan–Meier analyses. Multivariate Cox regression analyses were applied to further identify the prognostic effect of the biomarkers. In group comparisons of categorical variables, chi-square test was used. The analyses were carried out via GraphPad Prism (version 8.0) and R (version 4.2.3).

## Results

### In-depth profiling of the plasma proteome in cancer patients receiving anti-PD1 therapy

**Fig. 1:**
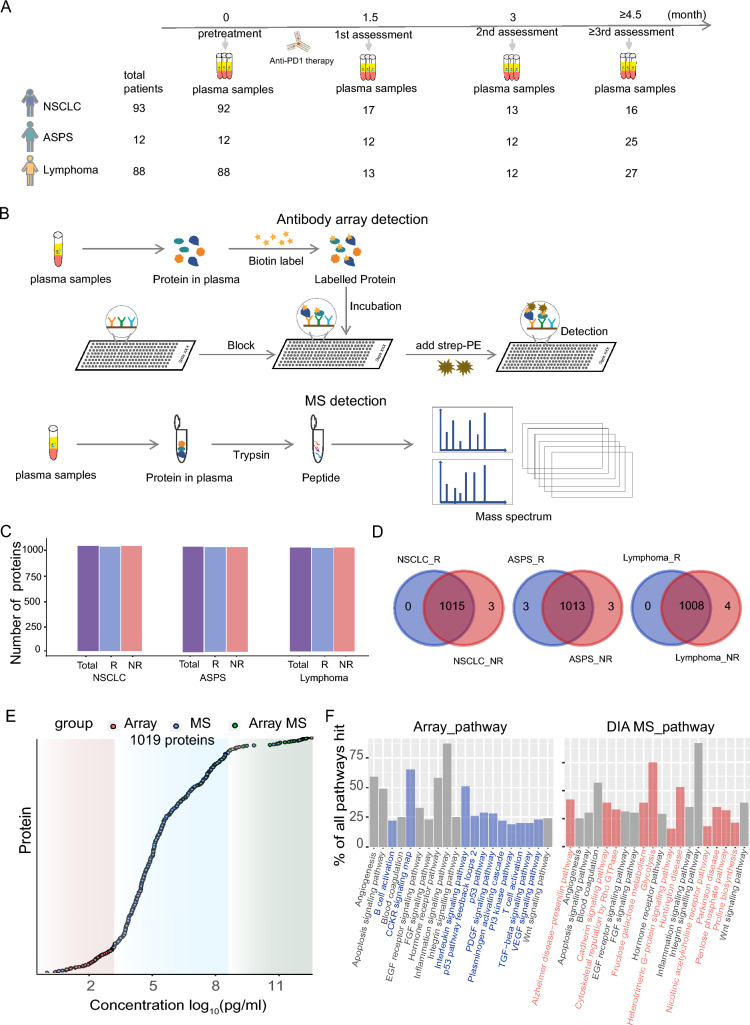
In-depth plasma proteomic profiling of cancer patients treated with anti-PD1 therapy. **A** Sample collection. A total of 339 plasma samples before and after anti-PD1 therapy from 93 NSCLC (92 patients have pre-treatment samples), 12 ASPS, or 88 lymphoma patients were collected. **B** The detection workflow of antibody array and MS method. **C** Total number of detected proteins in the R and NR groups of NSCLC, ASPS, and lymphoma cohorts. **D** Venn diagrams show the overlap of proteins in the R and NR groups of the NSCLC, ASPS, and lymphoma cohorts. **E** The concentration of plasma proteins identified using an in-depth proteomic platform. A total of 1019 proteins were identified, and the orange, blue, green plots indicate proteins identified by arrays, MS and array MS both, respectively. **F** Comparison of signal pathways of proteins identified by antibody microarrays and DIA-MS. Pathways in gray were shared by array and MS technology, and pathways in blue and orange indicate the unique pathways identified by array and MS technology, respectively

The plasma samples from NSCLC, ASPS, and lymphoma patients were collected before and at various time points after treatment (Fig. 1A). The plasma proteome was detected by DIA-MS and antibody microarray as previously described [19] (Fig. 1B). The detection correlation coefficients of plasma proteins within and among different experiments using antibody microarray were 0.976 and 0.913, respectively (Figure S1). To assess the reproducibility of DIA-MS, we employed H293 cell lysates as a control, which was tested in 45 consecutive time points throughout the plasma screen. The average Pearson correlation coefficient of 45 QC tests was 0.94 (Figure S2). These results suggest that our in-depth proteomic platform in plasma proteome screening is reproducible.

Using our platform, 96.5% (615/637, Figure S3) of the proteins were detected by microarray. We identified 1018, 1019, and 1012 proteins in NSCLC, ASPS, and lymphoma patients, respectively. Notably, there were no marked differences between the number of proteins identified in the R and NR patient groups in the three cancers (Fig. 1C, D). The concentration of these proteins was distributed across 12 orders of magnitude in plasma (Fig. 1E). Bioinformatics analysis showed that the proteins detected by antibody microarrays enriched B cell and T cell activation; CCKR, P53, PI3K, interleukin, PDGF, TGF-β, and VEGF pathways; and the proteins detected by DIA-MS enriched the cadherin, cytoskeletal regulation, proline biosynthesis, nicotinic acetylcholine, fructose galactose metabolism, and pentose phosphate pathways (Fig. 1F). These results demonstrate the advantages of our platform in acquiring the expression information of plasma proteins according to breadth (> 1000 proteins) and depth (12 orders of magnitude).

### Proteomic landscape mapping of the dysregulated pathways in anti-PD1-resistant cancer patients

**Fig. 2:**
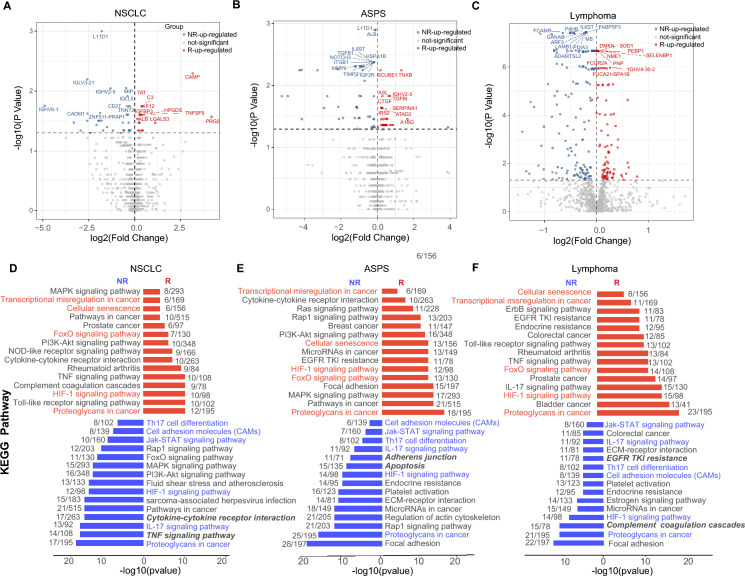
Different pre-treatment proteome profiles in the R and NR groups in three cancers. **A**–**C** Volcano plot of plasma proteins identified by in-depth proteomic detection in the NSCLC, ASPS, and lymphoma discovery cohorts. The black dotted line means FDR-adjusted *p* = 0.05, points above the line stand for differentially expressed proteins, and red and blue indicate proteins upregulated in R and NR patients, respectively. The top 10 proteins were shown in the figures. **D**–**F** Top KEGG pathways of differentially expressed proteins in the R and NR groups from the NSCLC, ASPS, and lymphoma discovery cohorts. Pathways marked in red and blue indicate common pathways among R or NR patients of three cancers, respectively. Pathways marked in bold italic indicate distinct pathways among NR patients of three cancers

Using the Mann–Whitney U test, 50 (NR 32 *vs*. R 18), 206 (NR 172 *vs*. R 34), and 268 (NR 179 *vs.* R 89) differentially expressed proteins were identified in NSCLC, ASPS, and lymphoma patients, respectively (Fig. 2A–C). Principle component analysis (PCA) revealed that the plasma profile in R group was distinct to that in NR patients (Figure S4). GO terms of these DEPs were enriched in processes of cytokine secretion and angiogenesis regulation in NSCLC, cell-substrate adhesion and metabolism in ASPS, and protein activation and humoral immune response in lymphoma. Moreover, some common processes were shared in NR patients of the three cancers, such as platelet degranulation (Figure S5). KEGG pathway analysis revealed that proteins upregulated in NR patients were enriched in cytokine-cytokine receptor interactions and TNF signal pathways in NSCLC (Fig. 2D), adherent junction and apoptosis in ASPS (Fig. 2E), and EGFR TKI resistance and complement coagulation cascades in lymphoma patients (Fig. 2F). Some common pathways were shared in NR patients compared to R patients across three cancer types, including immune-related pathways such as Th17 cell differentiation, the IL-17 signal pathway, and the HIF-1 signal pathway, as well as tumor, infection, autoimmune disease-related pathways such as the JAK-STAT signal pathway and cell adhesion pathways (Fig. 2D–F). For R patients, proteins altered might be involved in cellular senescence and transcriptional misregulation pathways (Fig. 2D–F). The activated pathway details are shown in Figure S6. Some differentially expressed proteins in cell adhesion (CADM1 in NSCLC, TUBA1B and ITGA5 in ASPS, and CDH5 and ICAM3 in lymphoma) and immune modulation (CCR3 and CD27 in NSCLC, FOS and MMP2 in ASPS, and CXCL13 and CCL26 in lymphoma) are shown in Figure S7.

### Longitudinal changes of the plasma proteome of cancer patients after therapy

**Fig. 3:**
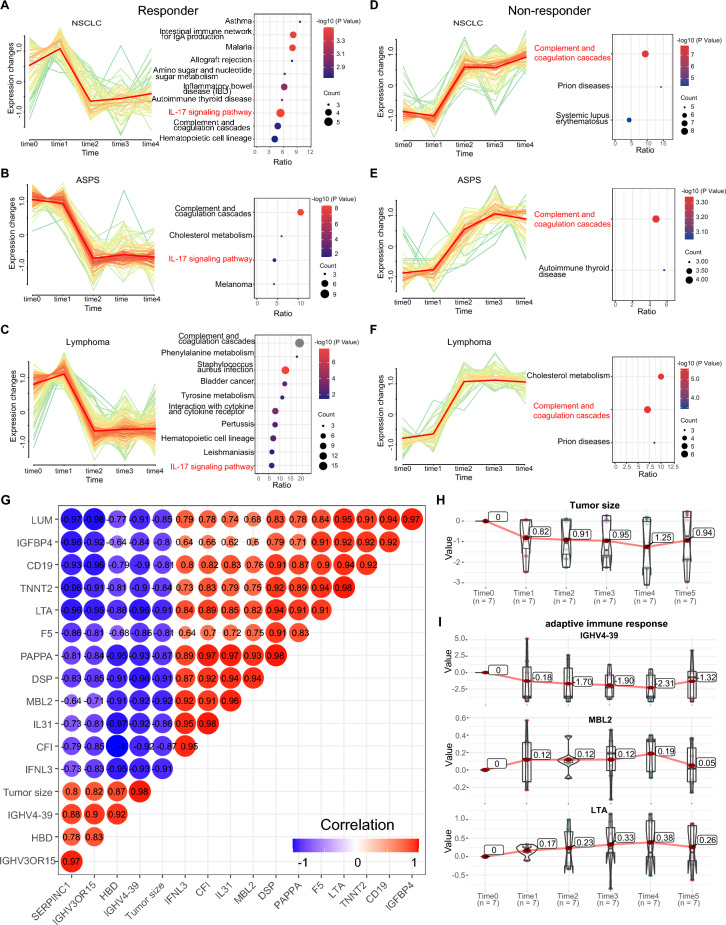
Dynamic changes in proteomic expression during anti-PD1 therapy. **A**–**C** Decreased protein cluster and corresponding signal pathway in NSCLC, ASPS, and lymphoma R patients. The signaling pathway marked in red is the same pathway shared among the three cancer types. **D**–**F** Increased protein cluster and corresponding signal pathways in NSCLC, ASPS, and lymphoma NR patients. The signaling pathway marked in red is the same pathway shared among three cancer types. **G** Correlation analysis of 15 proteins with tumor size, the numbers indicate correlation coefficient R, red indicates a positive correlation, and blue represents a negative correlation. **H** The dynamic changes in tumor size in patients who cancer had progressed following treatment. **I** Changes in the expression level of IGHV4-39, MBL2, and LTA during treatment

We performed longitudinal analyses of proteomic changes before and after anti-PD1 therapy, wherein we identified 339, 462, and 800 overexpressed proteins in the R group and 354, 201, and 258 overexpressed proteins in the NR group of NSCLC, ASPS, and lymphoma patients, respectively (*p* < 0.05). The DEPs were classified into six patterns by the Hiplot platform, and the pathways of each cluster were annotated by ClueGo in Cytoscape. The corresponding pathways for every cluster in the R and NR groups of NSCLC, ASPS, and lymphoma patients are shown in Figures S8–S10, respectively. Downregulated proteins after treatment in R were significantly enriched in the IL-17 signal pathway, whereas upregulated proteins were enriched in the vitamin digestion and absorption pathway (Fig. 3A–C and S11). For NR patients, proteins involved in the complement and coagulation cascades pathway were upregulated after treatment (Fig. 3D–F). These findings imply that the dysfunction of IL-17 signal modulation and coagulation function probably related to the benefit of anti-PD1 therapy.

Notably, we identified 15 noninvasive monitoring biomarkers whose expression is well correlated with changes in tumor size in the three cancers (R > 0.8, *p* < 0.05) (Fig. 3G). GO enrichment analysis further identified six proteins (CD19, CFI, IGHV3OR15-7, IGHV4-39, LTA, MBL2) related to adaptive immune response (*p* < 0.05). Longitudinal analysis showed that the expression of IGHV4-39 possessed a similar trend, while MBL2 and LTA showed an opposite trend to tumor size, which may have the potential to predict cancer recurrence after therapy (Fig. 3H, I).

### Identification of combinatorial targets in cancer patients who did not benefit from anti-PD1 therapy

**Fig. 4:**
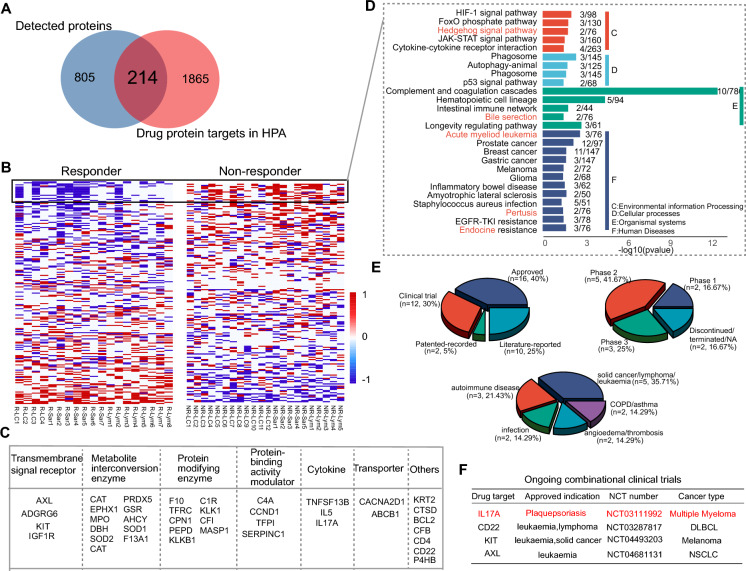
Identification of combinatorial drug targets with anti-PD1 therapy. **A** Venn diagram of current protein targets from the database and detected proteins in this study. **B** Heat map of protein targets during anti-PD1 treatment. The red and blue represent increased and decreased abundance of proteins following treatment, respectively. **C** Protein classes of 40 potential drug targets. **D** Pathway analysis of 40 potential drug targets. **C**–**F** indicate environmental information processing, cellular process, organismal systems, and human disease. **E** The pie charts of 40 targets with approved drugs or in clinical trial. **F** Ongoing clinical trials from identified combinatorial drug targets

To identify protein targets for cancer patients treated with anti-PD1 antibodies, we performed Venn diagram analysis of 1019 proteins that were detected using our in-depth plasma proteomics platform and 2080 protein targets from the HPA database (https://www.proteinatlas.org/). 214 proteins including 128 approved and 86 potential targets were identified (Fig. 4A). We further selected 40 potential targets whose expressions were upregulated in NR patients or downregulated in R patients among the list of all DEPs (Fig. 4B). Protein class analysis indicated these proteins belonged to signal receptor, enzyme, activity modulator, cytokine, and other classes (Fig. 4C). KEGG pathway analysis showed that these 40 proteins significantly enriched in cancer signaling pathways, including the HIF-1, JAK-STAT, cytokine–cytokine receptor interaction, and p53 signal pathways (Fig. 4D).

Detailed information on these targets, including full name, bioclass, drug information, and indications, is shown in Table S3. Among these protein targets, 70% (28/40) of the targets were approved or in clinical trials, and 66% (8/12) of these were administered in combination in clinical trials and have entered phases II and III. The approved indications focused on tumor/leukemia, autoimmune disease, or infectious disease (Fig. 4E). Notably, four clinical trials on myeloma, diffuse large B cell lymphoma (DLBCL), melanoma, and NSCLC are ongoing which combine anti-PD1 antibody and IL-17A (NCT03111992), CD22 (NCT03287817), KIT (NCT04493203), or AXL (NCT04681131), respectively (Fig. 4F).

To explore potential combination therapy mechanisms of anti-IL-17A and immunotherapy, analysis of the IL-17 signal relevant data showed high expression of multiple molecules on the pathway including MAPKs, IL6, CXCL8, MMP9, etc. The immune infiltration showed IL-17A was positive correlated with expression microphage M2, PD-L1, T cell regulatory (Tregs), negatively correlated with macrophage M1, in general, creating an immunosuppressive microenvironment (Figure S12). The therapeutic benefits of these combinatorial therapies to cancer patients have yet to be identified.

### Validation of predictive biomarkers in the independent lymphoma cohort and external NSCLC cohort

**Fig. 5:**
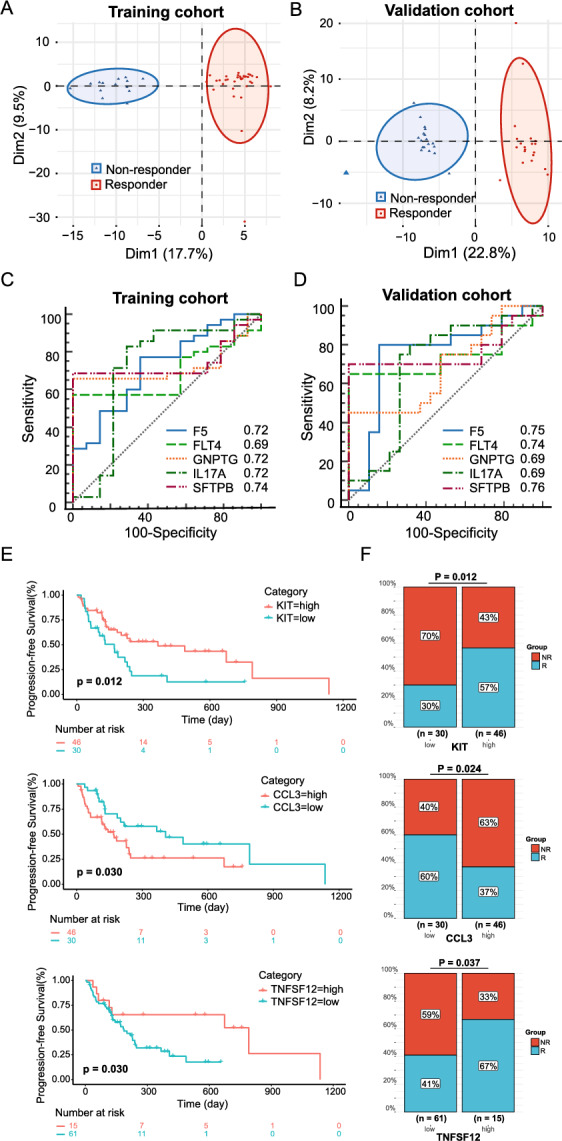
Validation of signaling pathways and predictive biomarkers in lymphoma and NSCLC cohorts. **A** PCA of R and NR patients using identified differentially expressed proteins in the lymphoma discovery cohort. **B** PCA of R and NR patients using validated differentially expressed proteins in the lymphoma validation cohort. **C**, **D** ROC analysis of five validated predictive biomarkers in the lymphoma discovery and validation cohort. **E** Kaplan–Meier curve of median PFS of ICI treatment in KIT, CCL3, and TNFSF12 high and low expression groups in external NSCLC validation cohort. **F** R and NR patients comparison in KIT, CCL3, and TNFSF12 high and low expression groups

To validate our findings, we collected pre-treatment samples from a validation cohort of 39 lymphoma patients, including 20 R and 19 NR patients. The results showed that 110 of 268 differentially expressed proteins in the lymphoma discovery cohort were successfully validated (*p* < 0.05). PCA analysis using the discovery and validation cohorts showed that patients with different responses were distributed in two separate areas (Fig. 5A, B). Pathway analysis showed that the validated proteins significantly enriched immune-related pathways, including the Th17 cell differentiation, IL-17 signal pathway, and estrogen signal pathways (Figure S13).

To identify specific predictive biomarkers, we selected five biomarkers (F5, FLT4, GNPTG, IL-17A, and SFTPB) that were differentially expressed between the R and NR patient groups in both lymphoma cohorts from the top 20% of significantly differentially expressed proteins (*p* < 0.05). The AUCs of these five biomarkers (F5, FLT4, GNPTG, IL-17A, and SFTPB) were 0.72, 0.69, 0.72, 0.72, and 0.74 in the discovery cohort and 0.75, 0.74, 0.69, 0.69, and 0.76 in the validation cohort, respectively (Fig. 5C, D). The results indicate that these biomarkers may potentially be utilized in predicting anti-PD1 therapy outcomes, though they still require validation in a large independent cohort.

To further validate the predictive capability of the biomarkers in NSCLC patients, we collected an external validation cohort of 76 pre-treatment NSCLC patients, including 33 R and 43 NR. Three (TNFSF12, CCL3, and KIT) biomarkers were observed to be effective in clinical validation. In KIT and TNFSF12 high expression group, significantly prolonged PFS (KIT: *p* = 0.012; TNFSF12: *p* = 0.030) and higher ratio of responder patients toward anti-PD1 therapy (KIT: 57% vs. 30%, *p* = 0.012; TNFSF12: 67% vs. 41%, *p* = 0.037) were observed. In CCL3 high expression group, patients presented a worse PFS (*p* = 0.030) and the proportion of non-responder patients was higher (60% vs. 37%, *p* = 0.024) compared to CCL3 low expression group (Fig. 5E, F). PD-L1 expression is a known predictive biomarker for anti-PD1 therapy in NSCLC; however, it is limited by the availability of biopsy tissue. In patients with PD-L1 expression available, the PD-L1 expression did not appear as a significant predictor of PFS and OS outcome in our cohort, and no association was observed between PD-L1 expression and three (TNFSF12, CCL3, and KIT) biomarkers (Figure S14). To avoid potential confounding factors, we did multivariate Cox regression analyses of the clinical characteristics including PD-L1 expression and the three plasma biomarkers. Multivariate Cox regression analyses confirmed that TNFSF12, CCL3, and KIT were independent risk factors (Figure S15).

## Discussion

Although extensive studies have been conducted in the field of immunotherapy, understanding of the molecular mechanism of anti-PD1 treatment of cancer remains unclear, and the availability of biomarkers for predicting responses remains limited. We conducted the largest, to our knowledge, dynamic proteomic study of 193 ASPS, NSCLC, and lymphoma patients who received anti-PD1 therapy using an in-depth plasma proteomic platform. And we validated the prognostic biomarkers in lymphoma (*n* = 39) and NSCLC cohort (*n* = 76).

The results identified a total of 534 DEPs, which involved in Th17 cell differentiation, IL-17 signaling pathway, JAK-STAT signaling pathway, and cell adhesion molecular pathways. Helper T cells 17 (Th17) are a subpopulation of T cells that are capable of secreting IL-17, IL-21, and IL-22, which mainly play important roles in autoimmunity and defense response against extracellular bacteria or fungi [29]. Th17 cell differentiation is induced by IL-23, STAT3, and retinoid acid-related-orphan nuclear receptorγt (RORγt). The activation of RORγt initiates the differentiation cascade of Th17 cells; following activation, RORγt promotes the expression of the IL-17 and IL-23 receptors (IL-23R) [30]. The JAK-STAT signaling pathway is necessary for immune modulation of host immune responses or immune interactions with non-immune factors [31, 32]. Besides Th17 cells, IL-17A can be secreted by natural killer T cells (NKT) and lymphoid tissue-inducing cells. IL-17 plays a role in promoting tumor development by facilitating angiogenesis and tumor cell proliferation and inhibiting apoptosis to promote tumor growth [33–35]. IL-23R can recruit Jak2, the JAK-STAT signaling system induced by IL-23 regulates Th17 cells, and drugs that inhibit the JAK-STAT signal pathway can target Th17 cells [36]. JAK-STAT signaling components are polarized in epithelial cells, and the JAK-STAT pathway also targets many genes controlling cell polarity and adhesion. Thus, activation of the JAK-STAT pathway is associated with cell adhesion [37]. The activation of JAK-STAT signaling is associated with upregulated PD-L1 expression in tumor cells, which promotes immune cell exhaustion and therapy resistance [38]. Consistent with the training cohort, we confirmed the Th17 and IL-17 signaling pathways in the validation cohort. We hypothesize that the immunosuppressive microenvironment induced by the JAK-STAT, Th17, and IL-17 axis correlates with efficacy of anti-PD1 therapy, although the detailed mechanism has yet to be elucidated.

The low efficiency of anti-PD1 therapy is currently an impeding problem that needs to be solved, and thus, screening for effective markers to predict and identify responder populations and to monitor cancer relapse is essential to improve the success of immunotherapy. To address this question, we validated eight plasma proteins (IL-17A, F5, FLT4, SFTPB, and GNPTG in lymphoma, TNFSF12, CCL3, and KIT in NSCLC) for response prediction and three plasma proteins (IL36G, SERPINC1, and LTA) for relapse monitoring. We found that the upregulated expression of IL-17A is associated with the poor prognosis of anti-PD1 therapy. In line with this finding, one study has reported that an increase in IL-17A can promote lung cancer growth by promoting inflammation, which results in resistance to anti-PD1 therapy and sensitizes tumors to cytokine and neutrophil depletion [39]. We also found that proteins to the IL-17 signal pathway were downregulated following treatment, which could lead to an anti-tumor microenvironment. Studies have shown that IL-17 reduces the presence of CD4 + and CD8 + lymphocytes in the tumor microenvironment, increases infiltrative Tregs, and further promotes angiogenesis, invasion, and metastasis [40]. In addition, coagulation factor V (F5) is the central regulator of hemostasis and results in the activation of prothrombin to thrombin. A study has reported the association between disorders of the coagulation–fibrinolysis system and immune activation by ICIs in cancer patients [41]. Fms-related tyrosine kinase 4 (FLT4) encodes vascular endothelial growth factor receptors (VEGFR3). This protein plays an important role in lymphangiogenesis and tumor metastasis. Studies also reveal that the VEGFR3 signal axis can influence tumor-associated microphages to inhibit anti-tumor immunity and promote tumor growth, and that it is correlated with patient survival [42, 43]. Besides efficacy, the association of blood proteins and toxicity of anti-PD1 therapy or use of corticosteroids is worthy of exploration.

Anti-PD1-resistant cancers require effective therapies, and the discovery of new drug targets or combinational drug targets to reverse anti-PD1 resistance has become a critical issue in the field of immunotherapy. To address this concern, we explored protein targets for combination therapy with anti-PD1 antibodies, which identified 40 potential drug targets that enriched cancer-related pathways. Among the 40 targets identified by longitudinal analysis, at least four clinical trials are ongoing, which is suggestive of its potential use. The IL-17 signaling axis plays an important role in the pathogenesis of rheumatoid arthritis, multiple sclerosis, and systemic lupus erythematosus. Antibodies targeting IL-17A have been approved for the treatment of plaque psoriasis; the observation in our cohort supports the rationale for combining anti-PD1 antibodies with IL-17A-targeted therapy in multiple cancer patients for overcoming immune suppression [44, 45]. A clinical trial utilizing the anti-IL-17A antibody alone or with the anti-PD1 antibody in multiple myeloma patients is ongoing [46]. In addition, the SERPINC1 gene encodes the antithrombin III protein, which was identified in our cohort to be correlated with tumor size and can serve as a monitoring biomarker; medicines targeting SERPINC1 have been approved for venous thrombosis and coagulation defect treatment, although the combinatorial effect of SERPINC1-targeted therapy with anti-PD1 antibodies has yet to be explored.

This study has several limitations. The number of ASPS samples employed in this study is limited, it would be ideal to involve more clinical samples and validate in a large and different cohort. In addition, the detailed roles of the biomarker proteins or function of combinatorial targets with anti-PD1 antibodies require further investigation, such as IL-17A, should be further elucidated or in vitro and in vivo experimentally validated. At last, PD-L1 expression did not appear as a significant predictor of outcome in multivariate analysis in our cohort, which may be due to the small subset of patients with available measured PD-L1 expression (*n* = 29), and the predictive performance of the proteins in combination with other known biomarkers (e.g., TMB, MSI/dMMR) can also be explored.

In conclusion, our study provides fundamental insights into the molecular changes in different cancer patients before and after anti-PD1 therapy and has identified biomarkers as well as combinatorial targets for patients who are resistant to treatment. These results are valuable for understanding the mechanism of anti-PD1 treatment and for developing new therapeutic strategies.

### Supplementary Information

Below is the link to the electronic supplementary material.

Supplementary file1 (DOCX 3741 kb)

## Data Availability

All data associated with this study are available in the main text or supplementary materials.
