# Supplementary material for: Longitudinal plasma proteomic analysis identifies biomarkers and combinational targets for anti-PD1-resistant cancer patients
Source: Cancer Immunol Immunother. 2024 Feb 13;73(3):47. doi: 10.1007/s00262-024-03631-7 (PMC10864508; doi:10.1007/s00262-024-03631-7)
Supplement: Supplementary file 1 — Supplementary file1 (DOCX 3741 kb) [file 262_2024_3631_MOESM1_ESM.docx]

**Supplementary materials for**

Longitudinal plasma proteomic analysis identifies biomarkers and combinational targets for anti-PD1-resistant cancer patients

**The PDF file includes:**

Figures S1 to S15

Tables S1 to S3

**Supplementary figure 1**


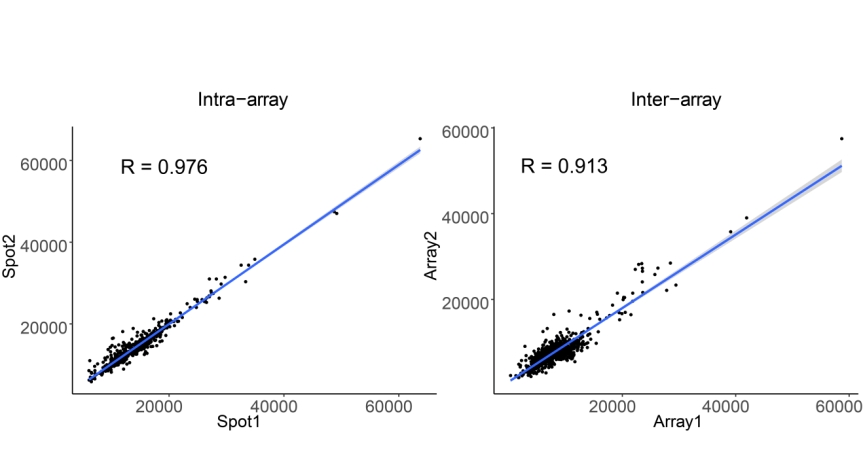


**Figure S1:** The reproducibility of the antibody array: Within- and between-array reproducibility.

**Supplementary figure 2**


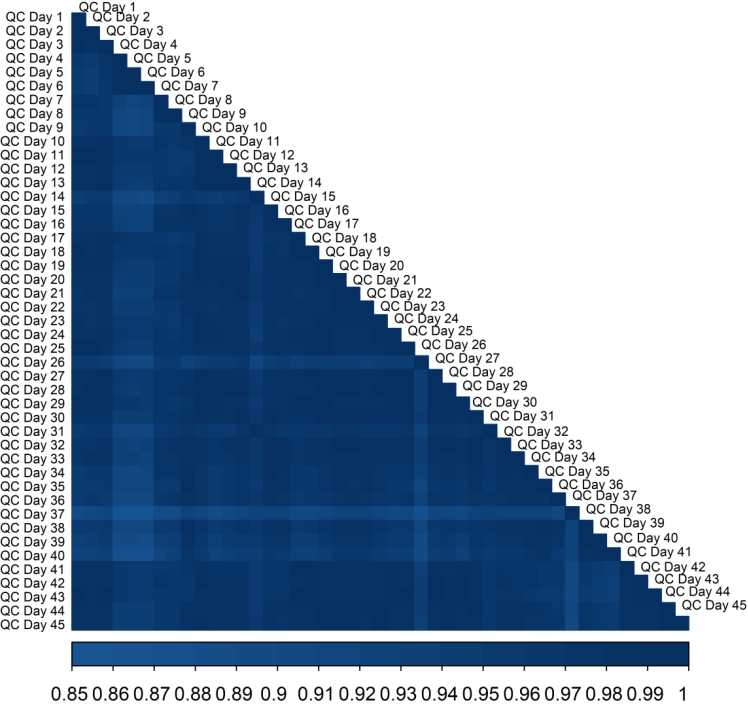


**Figure S2:** Person correlation of the same sample at different time points using the MS method.

**Supplementary figure 3**


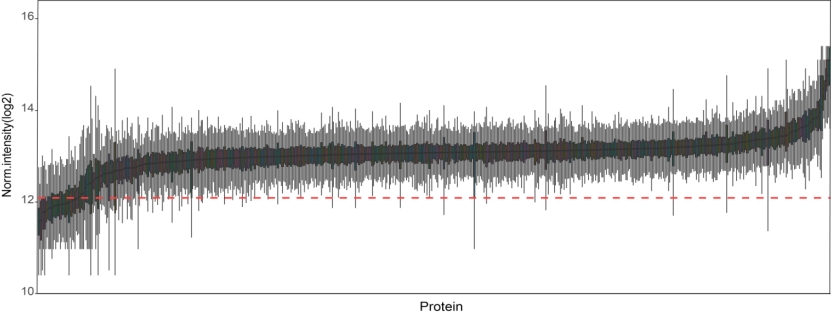


**Figure S3:** Normalized array signal distribution. The dotted line represents the mean of BSA as negative control. There are 615 proteins higher than the negative control and defined as positive detected proteins.

**Supplementary figure 4**


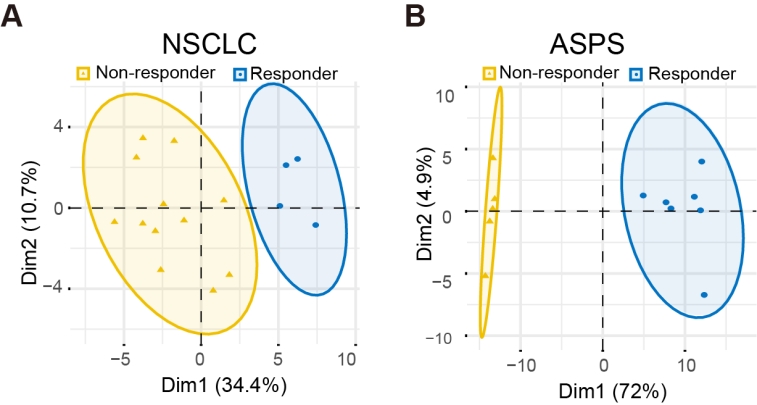


**Figure S4:** PCA of the R and NR patients using identified differentially expressed proteins in the NSCLC and ASPS cohorts.

**Supplementary figure 5**


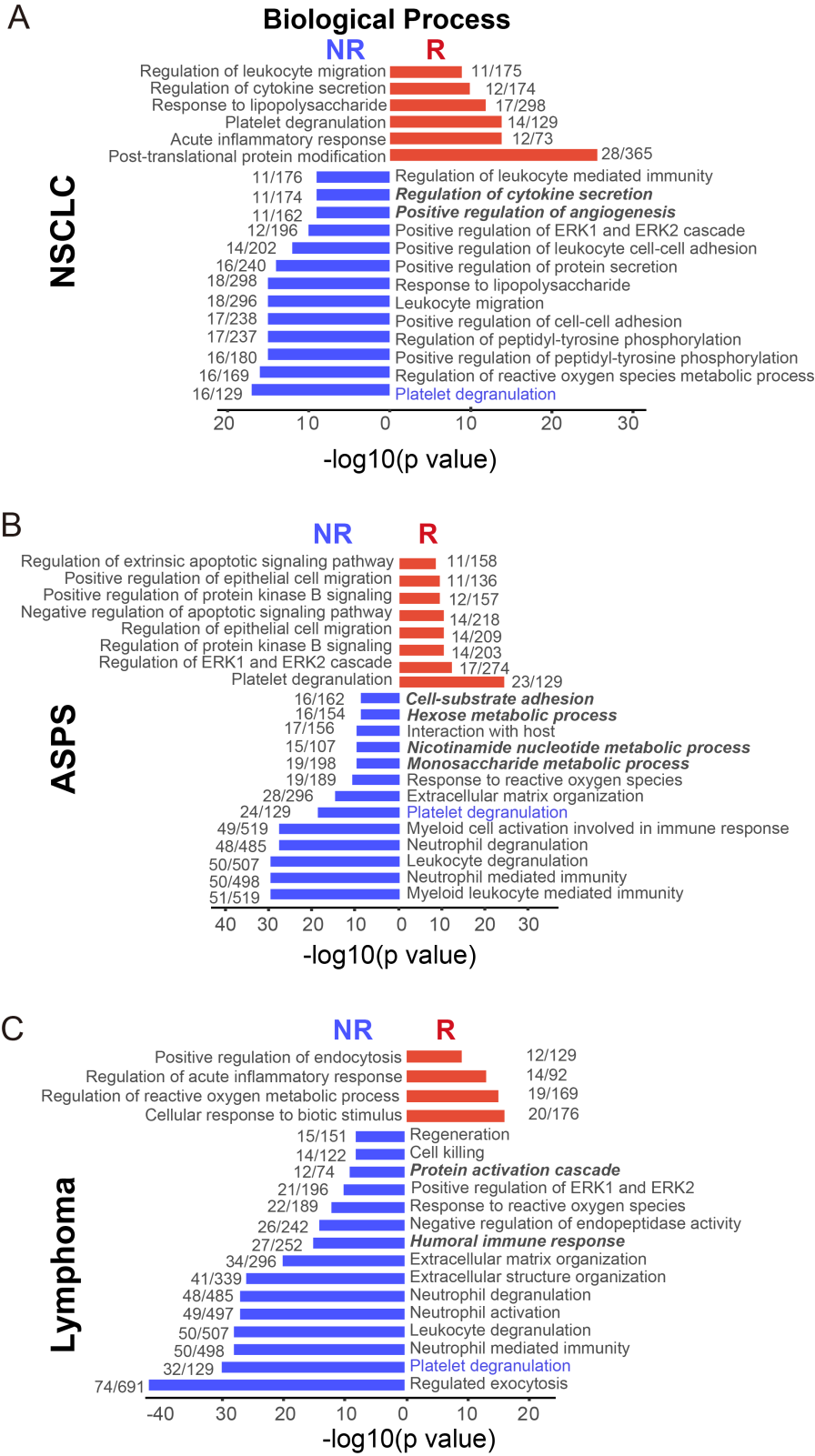


**Figure S5:** Top biological processes of differentially expressed proteins in the R and NR groups from the NSCLC, ASPS, and lymphoma discovery cohorts. Pathways marked in blue indicate common biological processes among NR patients of three cancers. Pathways marked in bold italic indicate distinct biological processes among NR patients of three cancers.

**Supplementary figure 6**
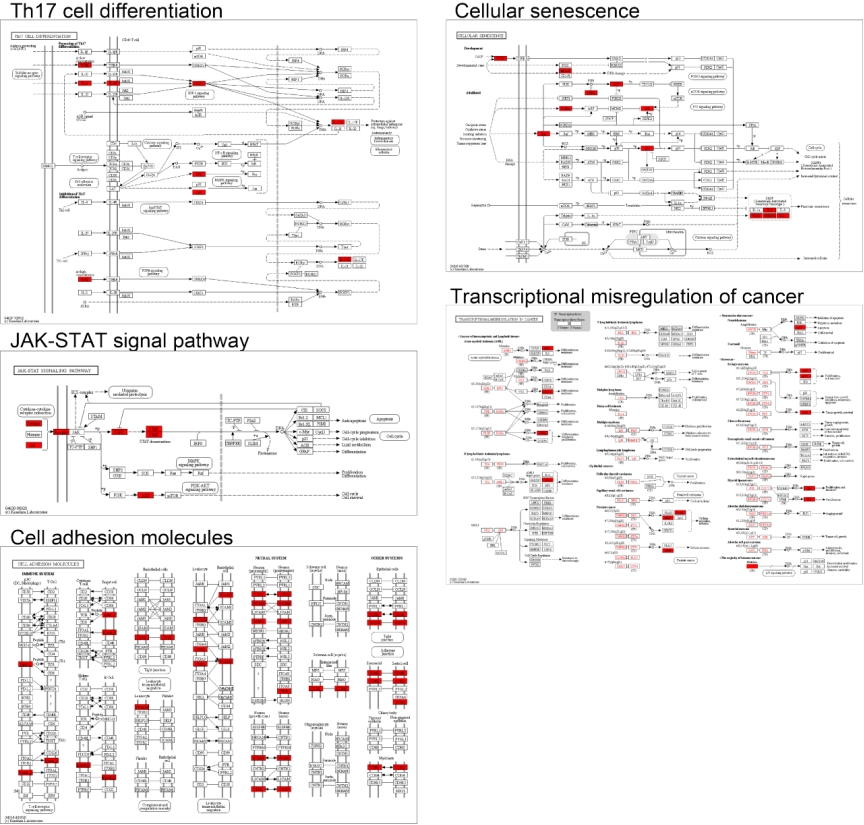
 **Figure S6:** The detailed signaling pathways activated in R and NR patients. The red boxes in the pathway indicate upregulated proteins.

**Supplementary figure 7**

**
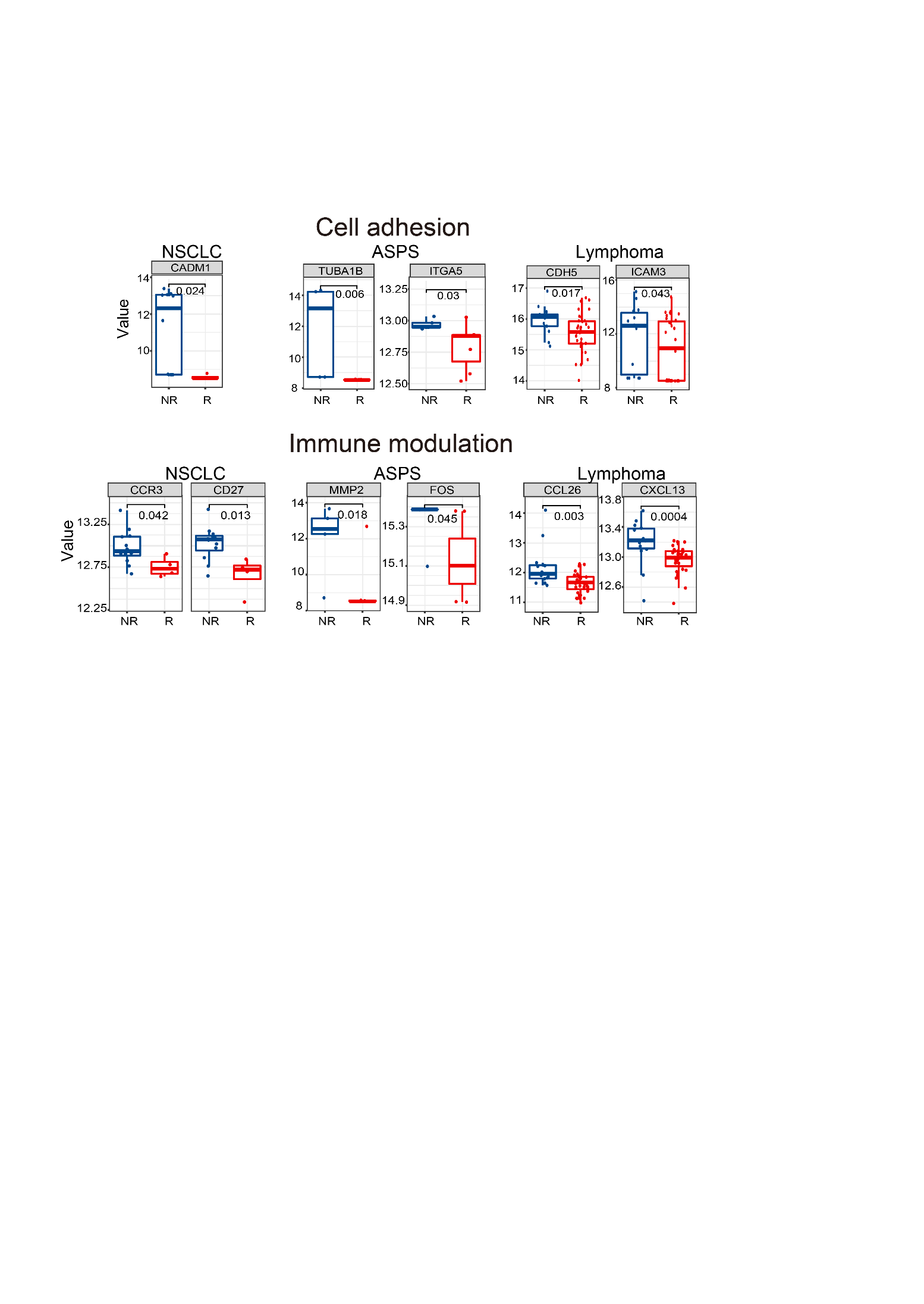
**

**Figure S7:** Box plot of example biomarkers that were related to cell adhesion and immune modulation.

**Supplementary figure 8**


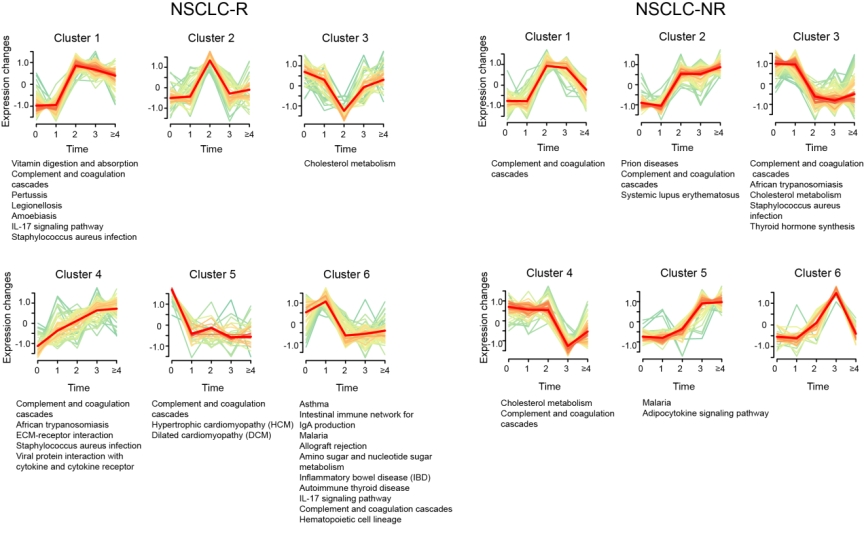


**Figure S8:** Protein expression clustering and corresponding enriched pathways following anti-PD1 treatment in R and NR NSCLC patients.

**Supplementary figure 9**


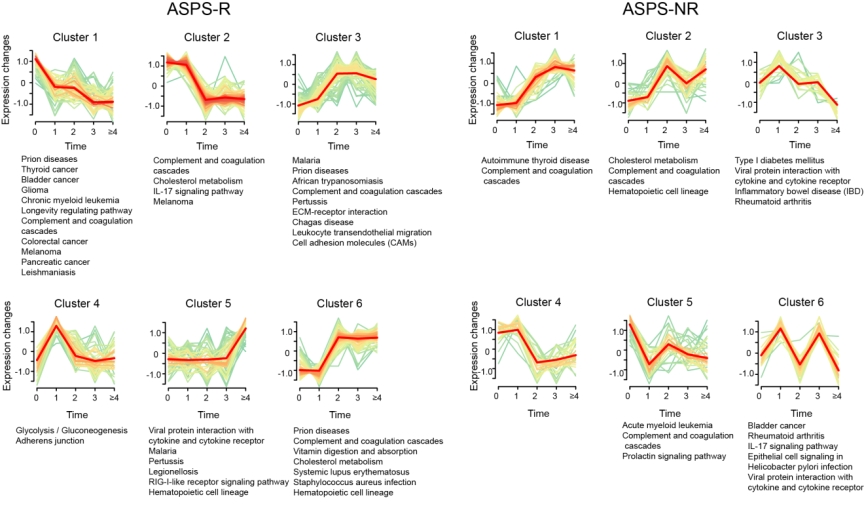


**Figure S9:** Protein expression clustering and corresponding enriched pathways following anti-PD1 treatment in R and NR ASPS patients.

**Supplementary figure 10**


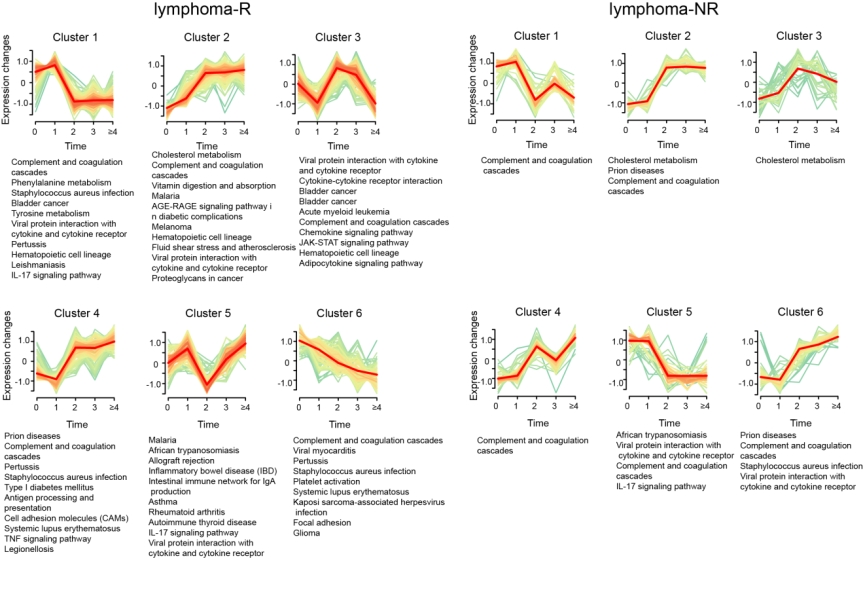


**Figure S10:** The protein expression clustering and corresponding enriched pathways following anti-PD1 treatment in R and NR lymphoma patients.

**Supplementary figure 11**


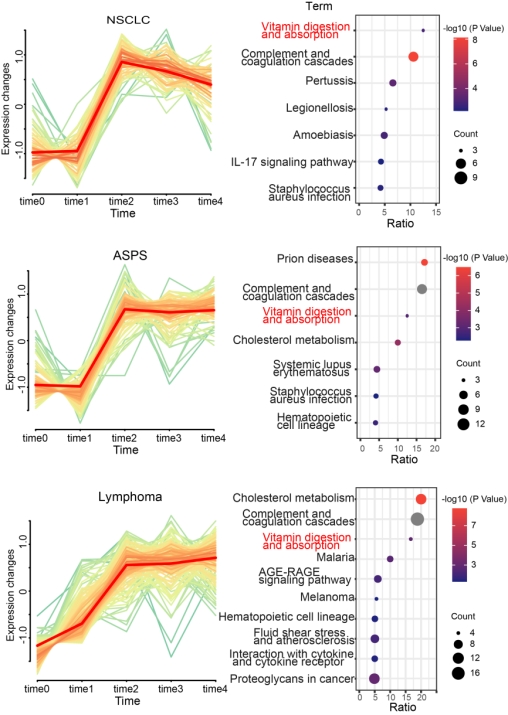


**Figure S11:** Increased protein clustering and corresponding signaling pathway in NSCLC, ASPS, and lymphoma R patients. The signaling pathway marked in red is the same pathway shared among the three cancer types.

**Supplementary figure 12**


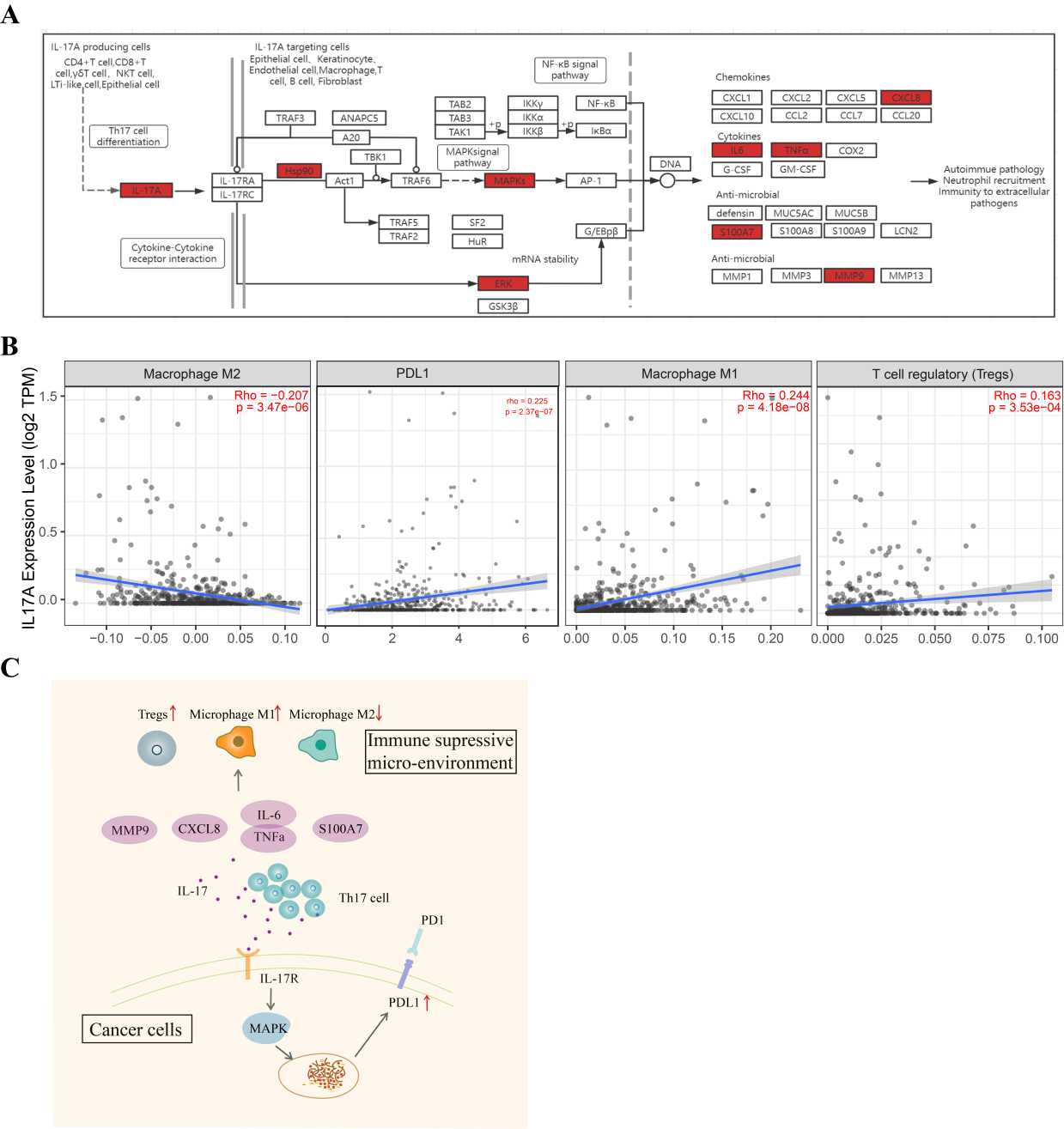


**Figure S12:** IL17A expression and association with immune microenvironment . (A)Upregulated proteins in IL17 signal pathway;(B) The association of IL17A with some immune microenvironment factors;(C) The probable mechanism of IL17A and the efficacy of immuno-therapy. Tregs:regulatory T cells

**Supplementary figure 13**

**
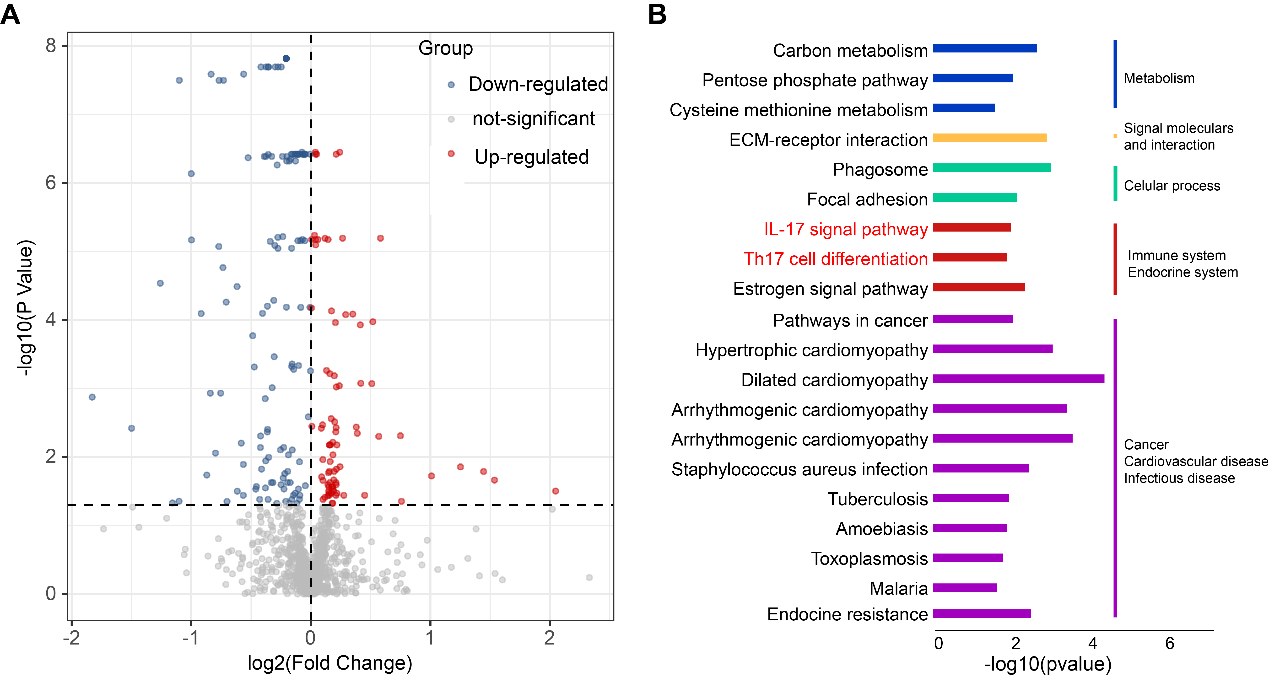
**

**Figure S13:** Volcano plot and KEGG pathway analysis of validated proteins. (A) Volcano plot of plasma proteins identified in the lymphoma validation cohort. The black dotted line means p=0.05, points above the line stand for differentially expressed proteins, and red and blue indicate proteins upregulated in R and NR patients, respectively.(B)KEGG pathways in the lymphoma validation cohort. The signaling pathway marked in red is the same pathway shared among the discovery and validation cohort.

**Supplementary figure 14**

**
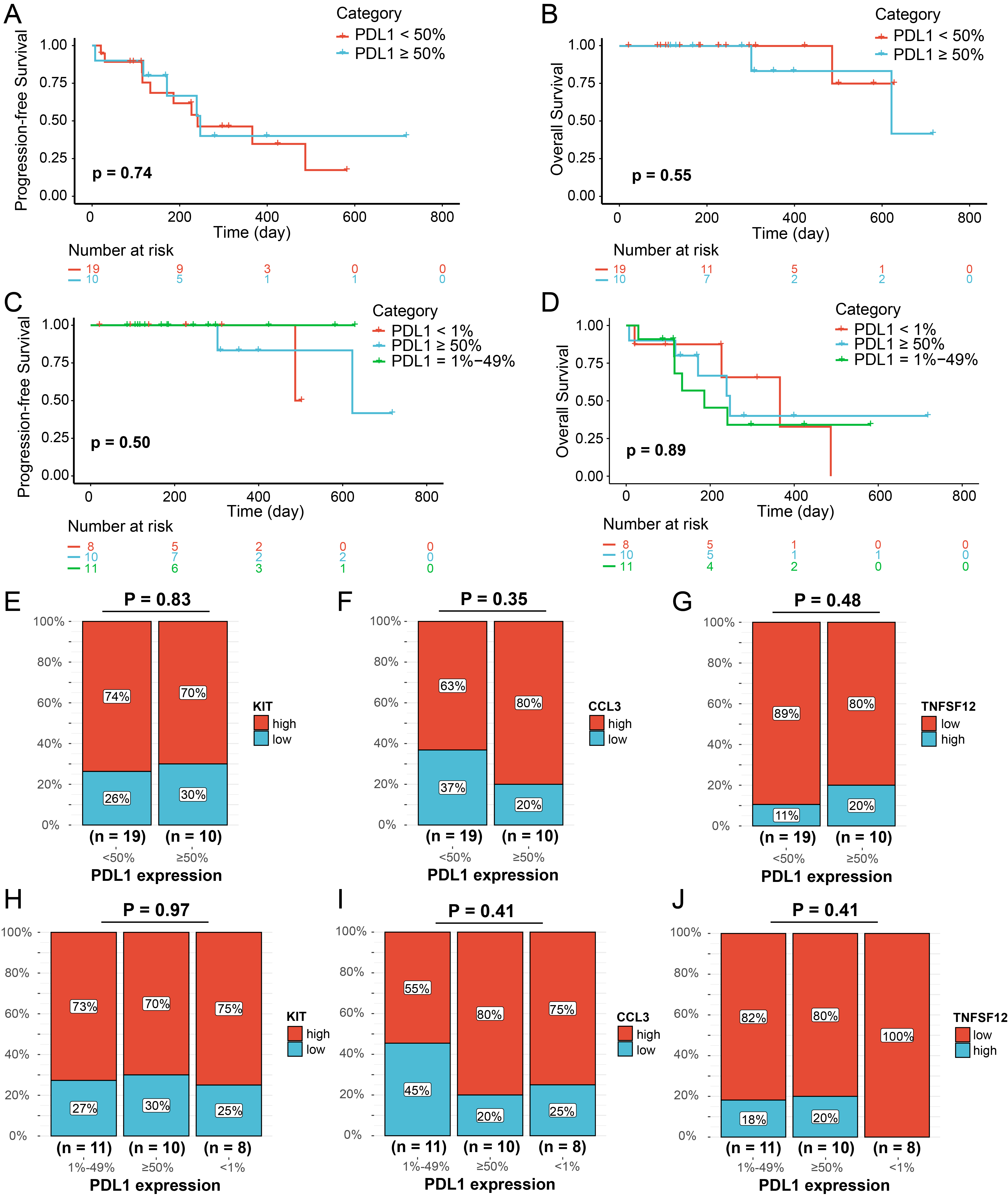
**

**Figure S14:** The prognostic value of PD-L1 and the association of PD-L1 expression with biomarkers. (A) (B) Kaplan-Meier curve of median PFS and OS in PD-L1 high and low expression groups (PD-L1 expression status: ＜50%, ≥50%). (C) (D) Kaplan-Meier curve of median PFS and OS in PD-L1 high and low expression groups (PD-L1 expression status: ＜1%, 1%-49%, ≥50%). (E) (F) (G) The association of PD-L1 expression with biomarkers (PD-L1 expression status: ＜50%, ≥50%). (H) (I) (J)The association of PD-L1 expression with biomarkers (PD-L1 expression status: ＜1%, 1%-49%, ≥50%).

**Supplementary figure 15**


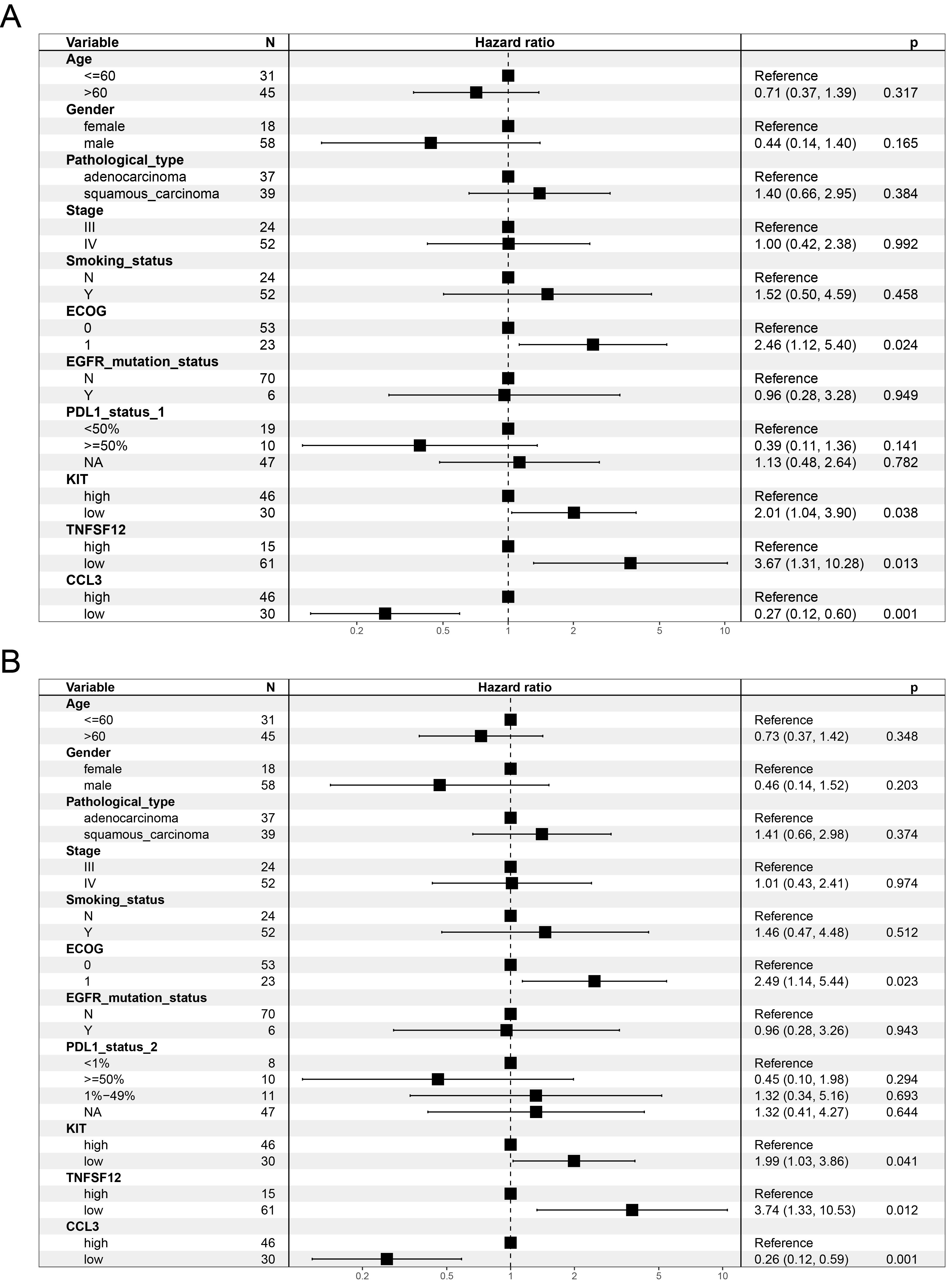


**Figure S15:** The prognostic value of KIT, CCL3, TNFSF12 and clinical characteristics in NSCLC. (A) Multivariate Cox regression analysis of KIT, CCL3, TNFSF12 and clinical characteristics (PD-L1 expression status: ＜50%, ≥50%). (B) Multivariate Cox regression analysis of KIT, CCL3, TNFSF12 and clinical characteristics (PD-L1 expression status: ＜1%, 1%-49%, ≥50%).

**Supplementary table 1:**

| **Cancer types** | Total patients | Pre-treatment | 1st evaluation | 2nd evaluation | 3rd evaluation | 4th evaluation | Total samples |
| --- | --- | --- | --- | --- | --- | --- | --- |
| **NSCLC** | 17 | 16 | 17 | 13 | 7 | 9 | 62 |
| **ASPS** | 12 | 12 | 12 | 12 | 9 | 16 | 61 |
| **Lymphoma discovery cohort** | 49 | 49 | 13 | 12 | 10 | 17 | 101 |
| **Lymphoma validation cohort** | 39 | 39 | 0 | 0 | 0 | 0 | 39 |
| **NSCLC validation cohort** | 76 | 76 | 0 | 0 | 0 | 0 | 76 |
| Total | 193 |  |  |  |  |  | 339 |

**Supplementary table 2**

| **Characteristic** | | **ASPS n=12 (%)** | **NSCLC n=17 (%)** | **Lymphoma discovery cohort**  **n=49 (%)** | **Lymphoma validation cohort**  **n=39 (%)** | **NSCLC**  **validation cohort**  **n=76 (%)** |
| --- | --- | --- | --- | --- | --- | --- |
| **Age (years)** | | | | | |  |
|  | Median | 33 | 60 | 32 | 33 | 63 |
|  | Range | 22-48 | 32-74 | 18-69 | 18-60 | 32-80 |
| **Gender** | | | | |  |  |
|  | Male | 5 (42) | 14 (82) | 28 (57) | 23 (59) | 58 (76) |
|  | Female | 7 (58) | 3 (18) | 21 (43) | 16 (41) | 18 (24) |
| **ECOG performance** | | | | |  |  |
|  | 0 | 4 (33) | 4 (24) | 31 (63) | 15 (38) | 53 (70) |
|  | 1 | 8 (67) | 13 (76) | 18 (37) | 24 (62) | 23 (30) |
| **Stage** | | | | |  |  |
|  | I | 0 (0) | 0 (0) | 0 (0) | 1 (3) | 0 (0) |
|  | II | 0 (0) | 0 (0) | 11 (22) | 7 (18) | 0 (0) |
|  | III | 0 (0) | 3 (18) | 5 (10) | 4 (10) | 24 (32) |
|  | IV | 12 (100) | 14 (82) | 32 (65) | 27 (69) | 52 (68) |
|  | Unknown | 0 (0) | 0 (0) | 1 (2) | 0 (0) | 0 (0) |
| **Clinical benefit** | | | | |  |  |
|  | Responder | 7 (58) | 5 (29) | 35 (71) | 20 (51) | 43 (57) |
|  | Non-responder | 5 (42) | 12 (71) | 14 (29) | 19 (49) | 33 (43) |
| ECOG: Eastern Cooperative Oncology Group | | | | |  |  |

| **Supplementary table 3:** | | |  |  |  |
| --- | --- | --- | --- | --- | --- |
| **genename** | **target name** | **target type** | **bioclass** | **drug information** | **Indications** |
| PRDX5 | Thioredoxin reductase (PRDX5) | Approved | Peroxide acceptor oxidoreductase | 1 Approved Drug:Motexafin gadolinium | Approved: Brain cancer |
| CD4 | T-cell surface glycoprotein CD4 (CD4) | Approved | Immunoglobulin | 1 approved drug: Ibalizumab 6 Clinical Trial Drugs: TNX-355(Phase 3),Zanolimumab(Phase 3),BT-061(Phase 2),et,al. | Approved:Human immunodeficiency virus disease Trial:Lymphoma,Asthma,Influenza virus infection,Solid tumour/cancer |
| CD22 | B-cell receptor CD22 (CD22) | Approved | Immunoglobulin | 4 approved:Inotuzumab ozogamicin;Moxetumomab pasudotox;Moxetumomab pasudotox;OspA lipoprotein. 34 clinical trial drugs:Epratuzumab(Phase 3),BL-22(Phase 2),CAR-T cells targeting CD22(Phase 2),et.al. | Approved:Lyme disease;Hairy cell leukaemia;Acute lymphocytic leukaemia. Trial:Acute lymphoblastic leukaemia,Non-hodgkin lymphoma,B-cell lymphoma,leukaemia,Haematopoietic/lymphoid cancer |
| TNFSF13B | B-cell-activating factor (TNFSF13B) | Approved | Cytokine: tumor necrosis factor | 1 approved drug:Belimumab. 2 Clinical Trial Drugs:Blisibimod(Phase 3),Tabalumab(Phase 3) | Approved: Systemic lupus erythematosus;Trial: Multiple myeloma |
| IL5 | Interleukin-5 (IL5) | Approved | Cytokine: interleukin | 3ApprovedDrugs:Benralizumab；Mepolizumab；Reslizumab | Approved: hypereosinophilic syndromes(HES）；eosinophilic granulomatosis with polyangiitis(EGPA)；Asthma |
| CACNA2D1 | Voltage-gated calcium channel alpha-2/delta-1 (CACNA2D1) | Approved | Voltage-gated ion channel | 6 Approved Drugs (Amlodipine, Diltiazem,Lercanidipine, et al.)；1 ClinicalTrialDrug:Imagabalin(Phase 3) | Approved:Chronicobstructivepulmonarydisease；Hypertension Trial:Generalized anxiety disorder |
| KRT2 | Keratin 2(KRT2) | Approved | cytoskeletal protein binding | 1 approved drug: Anthralin | Approved: psoriasis |
| IL-17A | Interleukin-17 (IL17) | Approved | Cytokine: interleukin | 2 approved drugs:Brodalumab；Ixekizumab;9clinicaltrialdrugs:Bimekizumab(Phase 3), ABT-122(Phase 2),CJM112(Phase 2),et ,al. | Approved:Plaquepsoriasis Trial:Rheumatoid arthritis,Acne vulgaris |
| ABCB1 | Multidrug resistance protein 1 (ABCB1) | Clinical trial | ABC transporter | 3Clinical Trial Drugs:CBT-1(Phase 3), EDP-322(Phase1),W-198(Phase1) | Trial:Non-small-cell lung cancer,Bacterial infection,Solid tumour/cancer |
| CCND1 | G1/S-specific cyclin-D1 (CCND1) | Clinical trial | intracellular proteins | 2Clinical Trial Drugs:ABT-263(Phase 2),Briciclib(Phase 1) | Trial:Relapsed or refractory chronic lymphocytic leukaemia,Solid tumour/cancer |
| SOD1 | Superoxide dismutase Cu-Zn (SOD Cu-Zn) | Clinical trial | Superoxide dismutase/reductase | 9Clinical Trial Drugs:Coprexa(Phase 1),ATN-224(Phase 2),Midismase(Phase 2),et,al | Trial:Neurological disorder, Solid tumour/cancer, Cerebral infarction, Interstitial lung disease,Myocardial infarction,Spinal cord injury, Dermatitis,Amyotrophic lateral sclerosis |
| DBH | Dopamine beta hydroxylase (DBH) | Clinical trial | Paired donor oxygen oxidoreductase | 2 Clinical Trial Drugs: Etamicastat(Phase 2),Nepicastat oral(Phase 2) | Trial:Heart failure,Post-traumatic stress disorder |
| CAT | Catalase (CAT) | Clinical trial | Peroxide acceptor oxidoreductase | 1 Clinical Trial Drug: EUK-189 (Phase 1) | Trial:Skin burns |
| TFPI | Tissue factor pathway inhibitor (TFPI) | Clinical trial | secreted proteins | 3 Clinical Trial Drugs:Tifacogin(Phase 3),BAX-499(Phase 1),NN-7415(Phase 1) | Trial: Sepsis;Factor IX deficiency |
| CTSD | Cathepsin D (CTSD) | Clinical trial | Peptidase | 1 Clinical Trial Drug: PMID10498202C1 | Trial: Multiple sclerosis |
| F13A1 | Coagulation factor XIIIA (F13A1) | Patented-recorded | Acyltransferase | Expert Opin Ther Pat. 2016;26(1):49-63. |  |
| MASP1 | Mannan binding lectin serine peptidase 1(MASP1) | Literature-reported | Enzymes | Front Immunol,. 2018 Aug 8;9:1851. |  |
| ADGRG6 | Adhesion G protein-coupled receptor G6(ADGRG6) | Literature-reported | G-protein coupled receptors | Genesis.2021 Mar 18;e23417. |  |
| CPN1 | Arginine carboxypeptidase (CPN1) | Literature-reported | Enzymes | Mol Immunol.2004 Jan;40(11):785-93. |  |
| AHCY | Adenosylhomocysteinase (AHCY) | Literature-reported | Ether bond hydrolase | Bioorg Med Chem. 2008 Apr 1;16(7):3809-15. |  |
| KIT | Tyrosine-protein kinase Kit (KIT) | Approved | Kinase | 7Approved Drugs:Imatinib,Pazopanib HCl,Ponatinib,et,al. 10 Clinical Trial Drugs: DCC-2618(Phase 3),PLX-3397(Phase 3),Famitinib(Phase 2),et,al. | Approved:Chronic myelogenous leukaemia,Acute lymphoblastic leukaemia, Renal cell carcinoma,Metastatic colorectal cancer,Thrombocytopenia Trial:Gastrointestinal stromal tumour,Alzheimer disease,Myelodysplastic syndrome,Solid tumour/cancer,Inflammation |
| IGF1R | Insulin-like growth factor I receptor (IGF1R) | Approved | Kinase | 2Approved Drugs: Mecasermin,Somatomedin-1. 15 Clinical Trial Drugs: OSI-906(Phase 3)，Rinfabate(Phase 2/3),AMG 479(Phase 2),et,al | Approved:Growth failure,Hormone deficiency.Trial:Breast cancer,Non-small-cell lung cancer,Graves ophthalmopathy,Diabetic nephropathy,Multiple myeloma,Colorectal cancer,Solid tumour/cancer |
| AXL | Tyrosine-protein kinase UFO (AXL) | Approved | Kinase | 1 Approved Drug:Gilteritinib 7 Clinical Trial Drugs:BGB-324(Phase 2),BI-505(Phase 2),MGCD265(Phase 2),et,al. | Approved:Acute myeloid leukaemia Trial:Breast cancer,Multiple myeloma,Non-small-cell lung cancer,Acute myeloid leukaemia,Solid tumour/cancer |
| BCL2 | Apoptosis regulator Bcl-2 (BCL-2) | Approved | B-cell lymphoma Bcl-2 | 4Approved Drugs: GDC-0199,MCI-186,Oral paclitaxel,Taxol.13 Clinical Trial Drugs:Oblimersen(Phase 3),RG7601(Phase 3),Liposomal encapsulated paclitaxel (LEP)(Phase 3)，et,al. | Approved:Chronic lymphocytic leukaemia,Amyotrophic lateral sclerosis,Breast cancer,Solid tumour/cancer Trial:Multiple myeloma, Chronic lymphocytic leukaemia,Breast cancer,Relapsed or refractory chronic lymphocytic leukaemia,Prostate cancer,Small-cell lung cancer,Follicular lymphoma,Psoriasis vulgaris |
| SERPINC1 | Antithrombin-III (ATIII) | Approved | Serpin protein | 5Approved Drugs:Ardeparin,Enoxaparin,Heparin Calcium,et,al. 9 Clinical Trial Drugs: Heparin low molecular weight(Phase 3),KW-3357(Phase 3),Unfractionated heparin(Phase 3),et,al | Approved:Deep vein thrombosis,Venous thrombosis,Coagulation defect,Tinnitus Trial:Thrombosis,Acute coronary syndrome,Asthma,Phlebothrombosis |
| F10 | Coagulation factor Xa (F10) | Approved | Peptidase | 11Approved Drugs:Apixaban,Certoparin sodium,Danaparoid,et,al. 15 Clinical Trial Drugs:PRT4445(Phase 3),Semuloparin(Phase 3),SSR-126517E(Phase 3)，et,al. | Approved:Thrombosis,Venous thromboembolism,Deep vein thrombosis,Haemophilia B,Atrial fibrillation,Factor VIII deficiency,Hematologic disease. Trial:Renal cell carcinoma,Bleeding disorder,Attention deficit hyperactivity disorder, Coagulation defect |
| C1R | Complement C1r subcomponent(C1R) | Approved | Blood group antigen proteins | 3 Approved Drugs: Palivizumab,Conestat alfa,Human C1-esterase inhibitor.2 Invetigational Drugs:Daclizumab,Gabexate. | Approved:Acute attack of hereditary angioedema, respiratory diseases casued by respiratory syncytial virus. Invetigational:Acute Pancreatitis |
| KLKB1 | Plasma kallikrein (KLKB1) | Approved | Peptidase | 2Approved Drugs:Ecallantide,Lanadelumab. 1 Clinical Trial Drugs:KVD001(Phase 1) | Approved:Retina venous occlusion,Hereditary angioedema.Trial:Diabetic macular edema |
| CFI | Complement factor I (CFI) | Clinical trial | Peptidase | 1 Clinical Trial Drugs:TP-20(Terminated) | Trial: Cerebrovascular ischaemia |
| MPO | Myeloperoxidase (MPO) | Clinical trial | Peroxidases | 3 Clinical Trial Drugs: E-101(Phase 3),AZD-3241(Phase 2), AZD4831(Phase 1) | Trial:Infectious disease,Parkinson disease, Heart failure |
| DBH | Dopamine beta hydroxylase (DBH) | Clinical trial | Paired donor oxygen oxidoreductase | 2 Clinical Trial Drugs: Etamicastat(Phase 2),Nepicastat oral(Phase 2) | Trial:Heart failure,Post-traumatic stress disorder |
| SOD2 | Superoxide dismutase Mn (SOD Mn) | Clinical trial | Superoxide dismutase/reductase | 1Clinical Trial Drug:Imisopasem manganese(Phase 2) | Trial: pain |
| CFB | Complement factor B (CFB) | Clinical trial | Peptidase | 1Discontinued Drugs: CAB-2(Discontinued in Phase 1) | Coronary artery disease |
| GSR | Glutathione reductase (GR) | Patented-recorded | Sulfur donor oxidoreductase | Expert Opin Ther Pat. 2017 May;27(5):547-556. | Patented: Asthma，Circulatory system disease，Indeterminate colitis |
| KLK1 | Tissue kallikrein (KLK1) | Literature-reported | Peptidase | URL: http://www.guidetopharmacology.org Nucleic Acids Res. 2015 Oct 12. pii: gkv1037. (Target id: 2865) | Respiratory system disease |
| EPHX1 | Epoxide hydrolase 1(EPHX1) | Literature-reported | Enzymes | Neurosci Biobehav Rev.2018 Apr;87:56-66. |  |
| TFRC | Transferrin receptor protein 1 (TFRC) | Literature-reported | Peptidase | J Blood Med. 2013 Mar 20;4:11-22. |  |
| P4HB | Prolyl 4-hydroxylase subunit beta(P4HB) | Literature-reported | Enzymes | Artif Cells Blood Substit Immobil Biotechnol.2009;37(1):1-12. |  |
| C4A | Complement C4a subcomponent(C4A) | Literature-reported | Blood group antigen proteins | Mol Psychiatry.2020 Jan;25(1):114-130. |  |
| PEPD | Xaa-Pro dipeptidase (PEPD) | Literature-reported | Peptidase | Rocz Akad Med Bialymst. 1998;43:201-9. |  |
